# Somatostatin receptor expression on von Hippel-Lindau-associated hemangioblastomas offers novel therapeutic target

**DOI:** 10.1038/srep40822

**Published:** 2017-01-17

**Authors:** Saman Sizdahkhani, Michael J. Feldman, Martin G. Piazza, Alexander Ksendzovsky, Nancy A. Edwards, Abhik Ray-Chaudhury, Dragan Maric, Marsha J. Merrill, Karel Pacak, Zhengping Zhuang, Prashant Chittiboina

**Affiliations:** 1 Surgical Neurology Branch, National Institute of Neurological Disorders and Stroke, National Institutes of Health, Bethesda, Maryland, USA; 2 Department of Neurosurgery, University of Virginia Health System, Charlottesville, Virginia USA; 3 Flow Cytometry Core, National Institute of Neurological Disorders and Stroke, National Institutes of Health, Bethesda, Maryland, USA; 4 Section on Medical Neuroendocrinology, National Institute of Child Health and Human Development, National Institutes of Health, Bethesda, Maryland, USA

## Abstract

Von Hippel-Lindau (VHL)-associated hemangioblastomas (VHL-HB) arise in the central nervous system (CNS), and are a leading cause of morbidity and mortality in VHL disease. Currently, surgical resection is the most effective way to manage symptomatic VHL-HBs. Surgically unresectable VHL-HBs or those in frail patients are challenging problems. Therapies targeting oncologic and vascular endothelial growth factor (VEGF) pathways have failed to demonstrate tumor control. Our experience and previous reports on VHL-HB avidity to somatostatin analogues suggested somatostatin receptor (SSTR) expression in VHL-HBs, offering an alternative therapeutic strategy. We explored this possibility by demonstrating consistent histologic expression of SSTR1, 2a, 4, and 5 in VHL-HBs. We found that somatostatin analogue octreotide induces apoptosis in VHL-HB stromal cells in a dose-dependent fashion by BAX – caspase-3 pathway unrelated to canonical VHL pathway. When administered to a patient with unresectable symptomatic suprasellar hemangioblastoma, octreotide resulted in tumor volume reduction, symptom stabilization, and tumor cytopenia on repeat ^68^Ga-DOTA-TATE positron emission tomography (PET) within 6 months, suggesting tumor infarction. We conclude that VHL-HBs harbor multiple SSTR subtypes that offer actionable chemo-therapeutic strategy for management of symptomatic, unresectable tumors by somatostatin analogue therapy.

Von Hippel-Lindau disease (VHL) is an autosomal-dominant tumor disorder affecting 1 in 36,000 live births^1^ due to mutations in the tumor suppressor *VHL* gene^2^. Up to 80% of patients with VHL develop central nervous system (CNS) hemangioblastomas (HBs) in the infratentorial regions, including the cerebellum and spinal cord, due to somatic ‘second hits’ at the VHL locus^3^,^4^. Approximately half of the VHL associated HBs (VHL-HBs) will grow over time and lead to mass effect-related neurological symptoms^5^. Surgical resection of symptomatic VHL-HBs remains the standard-of-care, but even with optimal management VHL-HBs remain a leading cause of death in VHL patients^3^,^4^. In some cases, surgical resection of symptomatic VHL-HBs is not an option due to unresectable locations or due to extensive co-morbidities. Development of chemotherapy and anti-angiogenic therapy for such patients remains underdeveloped due to the indolent nature of VHL disease^6^,^7^,^8^,^9^,^10^. Due to relatively long life expectancy of VHL patients^11^, treatment with toxic chemotherapeutic agents is not feasible for extended periods of time. Alternative strategies for long term management of unresectable VHL-HBs may arise from observations that VHL-HBs originate from hematopoietic precursors^12^,^13^, and that hematopoietic stem cells are known to express somatostatin receptors^14^.

Our experience with DOTA-TATE positron emission tomography (PET) imaging of VHL patients confirms previous reports of VHL-HB avidity to somatostatin analogues (Fig. 1A–D)^15^,^16^,^17^. However, the therapeutic significance of somatostatin receptor (SSTR) expression on VHL-HBs remains unknown. Neuroendocrine tumors that express upregulated SSTRs demonstrate a clinical response to somatostatin analogues^18^,^19^. In this study, we first demonstrate that VHL-HBs express multiple SSTRs subtypes including 1, 2a, 4, and 5. Then, we demonstrate an increase in apoptosis with SSTR stimulation in human HB stromal cells harvested from surgically resected VHL-HBs. We further confirm this novel strategy for management of surgically unresectable symptomatic VHL-HB in a single patient with administration of octreotide for up to 9 months.

## Results

### VHL-associated HBs express SSTR subtypes 1, 2a, 3, 4, and 5

**Figure 1:**
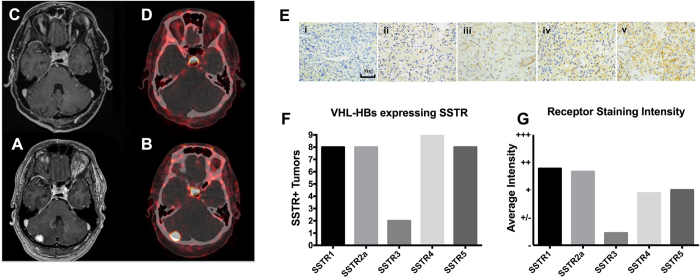
Von Hippel Lindau Disease related hemangioblastomas express somatostatin receptors. Patients with von Hippel Lindau disease (VHL) undergoing surgical resection of posterior fossa hemangioblastoma provide confirmation of DOTATATE avidity of these lesions. The post-operative T1 weighted MRI image (**A**) and DOTATATE PET image (**B**) confirm complete resection of a DOTATATE avid posterior fossa hemangioblastoma (**C**,**D**). Representative images (**E**) of anti-SSTR IHC staining classified as negative (i), positive/negative (ii), positive (iii), double positive (iv) or triple positive (v). Scale bar = 50 μm. Average staining intensity for each SSTR subtype is shown in panel F. Number of tumors with SSTR expression are categorized by receptor subtype (**G**). Positive staining patterns were found in 8/9 tumors against SSTR1, in 8/9 tumors against SSTR2a, in 2/9 tumors against SSTR3, in 9/9 tumors against SSTR4, and in 8/9 tumors in SSTR5.

Following optimization, 9 VHL-HBs were stained for SSTR subtypes 1, 2a, 3, 4 and 5, (Fig. 1E and Table 1). VHL-HBs demonstrated strong expression patterns for all receptor subtypes except SSTR3 (Fig. 1F). Specifically, 100% of tumors demonstrated positive SSTR4 expression and 89% of tumors demonstrated positive SSTR1, 2a, and 5 expression while only two tumors had SSTR 3 expression by IHC (Fig. 1G). Co-expression of more than one receptor subtype was seen in all tumors (100%), with all tumors showing positivity for at least 3 SSTR subtypes. Multiple SSTR receptor subtype expression may predict clinical response^20^. Additionally, both SSTR dependent imaging modalities (e.g. DOTA-TATE PET)^21^ and somatostatin analogues (e.g. octreotide)^22^ tend to be selective for SSTR subtype 2a. IHC results from additional tumors stained for <5 SSTR subtypes (n = 27), due to insufficient tissue, are available as Supplementary Table 1.

### VHL-HB cell viability is decreased with Octreotide exposure

**Figure 2:**
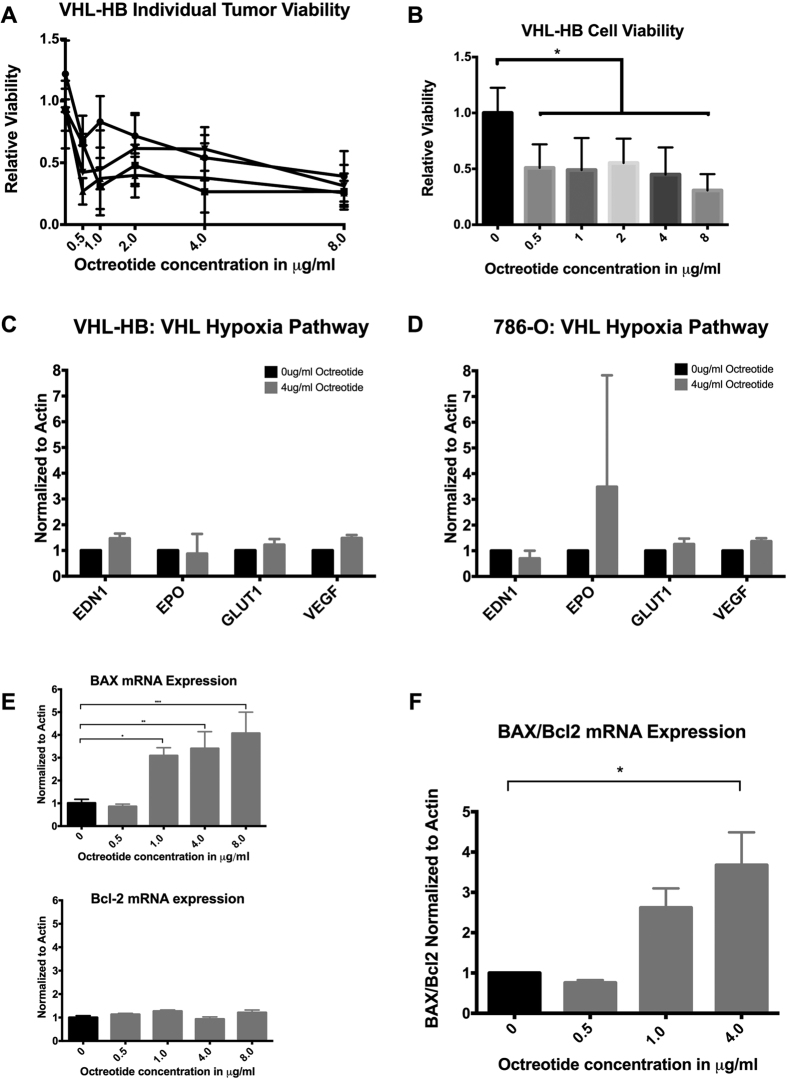
Primary culture of four VHL-HBs demonstrated dose-dependent decreased survival with Octreotide treatment. Four individual tumors were analyzed independently, with technical triplicates for each dose within each experiment (**A**). Doses of 0.5, 1, 2, 4, and 8 μg/ml all displayed significantly decreased cell survival with XTT assay (**B**). Statistically significant reduction in overall survival at 0.5, 1, 2, 4, and 8 μg/ml of octreotide was found, where there was a 59%, 50%, 49%, 58%, and 76% (one way ANOVA p = 0.0004), respectively. Octreotide does not affect canonical VHL pathway gene expression (**C**,**D**). VHL-HB stromal cells or 786-O cells were each incubated in 4 μg/ml octreotide for 48 hrs prior to mRNA extraction. Relative changes in the VHL-HIF pathway genes *EDN1, EPO, GLUT1*, and *VEGF* were assessed with RT-PCR. In both the VHL-HB stromal cells and the 786-O cells, there was no significant change in *EDN1, EPO, GLUT1* or *VEGF* expression. Octreotide induces a dose-dependent increase in BAX and Bcl2 expression levels (**E**,**F**). VHL-HB stromal cells were exposed to octreotide-acetate for 48 hrs and subsequently analyzed via RT-qPCR for BAX and Bcl-2 mRNA expression levels. Significantly increased BAX/Bcl-2 ratio was found at 4 ug/ml (4.144 ± 0.07914, p = 0.02, CI −4.58 to −1.70; one-way ANOVA, MD ± SE, p, 95% CI of difference). Results normalized to Actin.

To determine the sensitivity of VHL-HB stromal cells to Octreotide *in-vitro*, XTT cell viability assay was performed. Stromal cells from four surgically resected VHL-HBs were harvested and investigated for cell survival. We performed flow cytometry to confirm the presence of viable HB stromal cells in our experimental substrates. HB stromal cells were gated for marker carbonic anhydrase-IX (CA-IX)^23^, and cC3/cPARP positive cells were marked as apoptotic cells. We found satisfactory proportions of viable HB stromal cells (CA-IX positive, cC3/cPARP negative) cells with the harvesting procedure (60 ± 16%) (Supplementary Figure 1). Four different VHL-HB tumors were harvested into a stromal cell suspension and subsequently incubated for 48 hrs in 0, 0.5, 1, 4 and 8 μg/ml of octreotide-acetate (Fig. 2A). XTT cell viability analysis was conducted. All concentrations of octreotide uniformly induced a reduction in cell survival. At 0.5, 1, 2, 4, and 8 μg/ml there was a 59%, 50%, 49%, 58% and 76% statistically significant reduction in cell viability, respectively (one way ANOVA p = 0.0004) (Fig. 2B). These doses are considered consistent with serum-attainable concentrations in patients^24^.

### Canonical VHL pathway is not involved in somatostatin analogue mediated anti-tumor effect

To investigate the effects of octreotide on the canonical VHL pathway (pVHL- HIF – hypoxia pathway) for tumorigenesis^25^,^26^, we incubated VHL-HB stromal cells and *VHL* deficient (*VHL* −/−) 786-O cells^27^ with 4 μg/ml of octreotide for 48 hours, and assessed HIF pathway suppression by RT-qPCR^25^,^26^,^27^. In the VHL-HB stromal cells and 786-O cells, octreotide did not significantly suppress expression of genes endothelin-1 (*EDN1*), erythropoietin (*EPO)*, glucose transporter 1 (*GLUT1)*, or vascular endothelial growth factor (*VEGF)* implicated in the canonical VHL pathway (Fig. 2C). Similarly, in 786-O cells no change in *EPO, GLUT1, EDN1,* or *VEGF* expression (Fig. 2D). These findings suggest a mechanism other than the canonical VHL pathway leading to reduced VHL-HB cell survival with Octreotide exposure.

### Octreotide induces apoptosis in VHL-HB stromal cells

We investigated the BAX/Bcl-2 pathway as a mechanism of octreotide-induced apoptosis in VHL-HB stromal cells. In various types of malignancies such as breast and hepatocellular cancer, somatostatin has been noted for its induction of apoptosis through the direct pro-apoptotic factor BAX^28^,^29^. Further, increased BAX/Bcl-2 ratio has been shown in response to SSTR2 mediated apoptosis in pancreatic cancer^30^. Therefore, we conducted RT-qPCR in order to assess relative mRNA expression of pro-apoptotic marker BAX and anti-apoptotic regulator Bcl-2 (Fig. 2E,F). VHL-HB stromal cells were exposed to octreotide at 0, 0.5, 1, 4 μg/ml for 48 hrs. Compared to controls, the BAX/Bcl-2 mRNA levels exhibited a dose-dependent increase in expression (Fig. 2F). In accordance with previously described mechanisms of somatostatin-induced apoptosis, at 4 μg/ml of octreotide a statistically significant increase in the BAX/Bcl-2 ratio was found (4.144 ± 0.07914, p = 0.02, CI −4.58 to −1.70; one-way ANOVA, MD ± SE, p, 95% CI of difference).

### VHL patient treated with Octreotide for surgically unresectable HB

**Figure 3:**
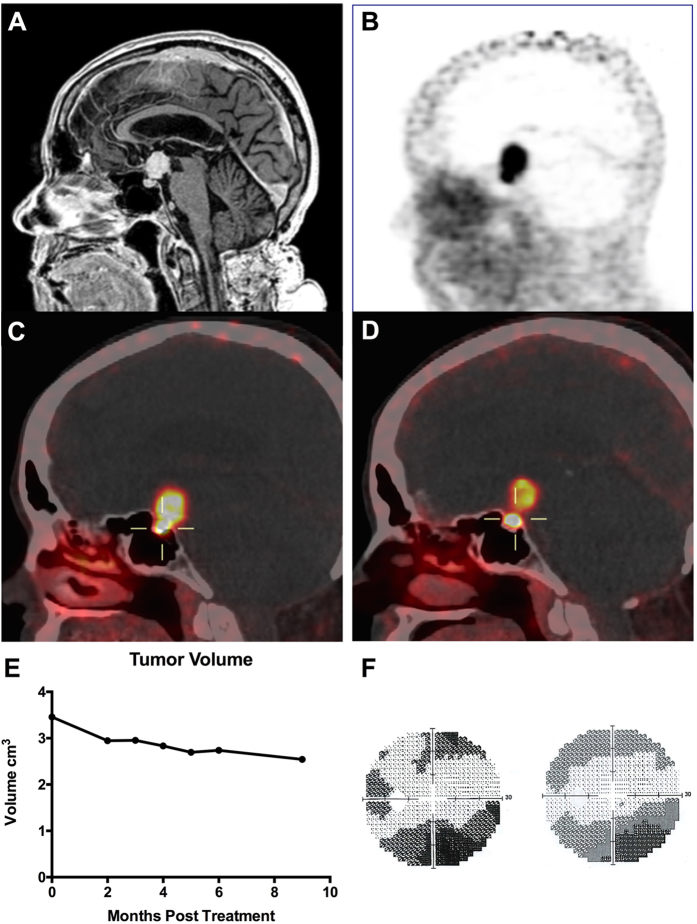
Symptomatic, surgically unresectable VHL-HB treated with octreotide. (**A**) T1 magnetic resonance image with contrast of 64 year-old female patient with germline *VHL* missense mutation presents with a 3.45 cm^3^ suprasellar HB. (**B**) ^68^GA-DOTATATE PET/CT imaging of suprasellar HB showing avid ^68^GA-DOTATATE uptake. (**C**) ^68^Ga-DOTATATE-PET imaging at presentation and 6 months after octreotide treatment (**D**). The pituitary stalk HB exhibits diminished uptake of 12.0 SUV compared to 22.6 SUV in initial study. Central photopenia also noted, indicative of necrotic process. (**E**) Tumor volume was measured using Osirix® software on FLAIR images. Seven images were analyzed, with the first image acquired just prior to initiation of Octreotide treatment. Measurements in cm^3^. (**F**) Visual field analysis of patient’s left visual field prior to starting octreotide treatment (left) and 6 months after beginning treatment.

A 64-year-old female with a germline *VHL* missense mutation (R167W), a known large suprasellar hemangioblastoma, and history of right eye enucleation presented with worsening vision of her remaining left eye. The patient had a past history of severe VHL disease with bilateral clear cell renal cell carcinomas, bilateral pheochromocytomas, pancreatic cysts, retinal HBs, and multiple CNS HBs. Neurological exam was significant for Romberg sign and mild dysmetria of bilateral upper extremities. Left eye extraocular movements and pupillary reaction intact. A visual field test of the left eye revealed new onset inferior temporal quadrantanopsia. Magnetic resonance imaging confirmed a slowly growing (3.45 cm^3^) suprasellar hemangioblastoma within the circle of Willis with increased optic tract edema and compression of the optic chiasm (Fig. 3A).

The newly symptomatic suprasellar mass was deemed inoperable due to the location (suprasellar, within the circle of Willis), her extensive co-morbidities, and the risk of loss of vision in her remaining eye with an attempted surgical resection. Whole body ^68^Ga-DOTATATE-PET revealed that the suprasellar mass was avid for DOTATATE (maximum standard uptake value (SUV_Max_) = 22.6) (Fig. 3B and C). As such, octreotide was initiated off label with a trial dosing of 20 μg daily subcutaneously for two weeks. Following this, she was prescribed long-acting Octreotide-LAR 30 mg by intramuscular injection, once a month, for 9 months. On Octreotide-LAR, she reported diarrhea managed with increased pancrealipase dosing and an increased insulin requirement. Follow up ^68^Ga-DOTATATE-PET imaging was done 6 months after primary scans and more than one month after her most recent octreotide dose. These images revealed maximum SUV_Max_ of 12.0, compared to the initial SUV_Max_ of 22.6 prior to starting treatment. Moreover, the 6 month follow up scan revealed central tumor photopenia, indicative of necrosis (Fig. 3D). As a reference, the pituitary uptake initially measured 24.9 SUV and 6 months after treatment measured 50.3 SUV. Serial FLAIR MRI images were acquired periodically throughout octreotide treatment and revealed a steady decline in the volume of the suprasellar mass from 3.45 cm^3^ to 2.54 cm^3^ with 9 months of Octreotide-LAR therapy (Fig. 3E). Furthermore, visual field analysis remained stable with continuous therapy (Fig. 3F).

## Discussion

In this report, we demonstrated that surgically resected VHL-HBs reliably express SSTR subtypes 1, 2a, 4, and 5, with maximal expression of SSTR subtypes 1 and 2a. This is consistent with the expression patterns of SSTR2 in various neuroendocrine tumors (NETs), such as gastrinomas, VIPomas, and glucagonomas^31^. Unlike what has been shown in sporadic RCC, octreotide treatment in our patient did not result in growth arrest of VHL-associated ccRCC tumors. Additionally, these tumors were not detected on DOTATATE imaging (Supplementary Figure 2) suggesting the lack of SSTR2 on VHL-associated ccRCC and that SSTR2 expression is specific to VHL-HB. SSTR2 expression has implications for both imaging and treatment of neuroendocrine tumors. Somatostatin radiotracers including DOTATATE have high affinity for SSTR2^21^. SSTR2 expression is also highly correlated with disease response (tumor volume and hormone secretion) in pituitary^32^,^33^ and other tumors^22^ following somatostatin analogue therapy. SSTR3 and SSTR4 are normally found throughout the brain^34^,^35^ but are less widely expressed in human cancers when compared with SSTR1, 2, and 5^36^,^37^. This is consistent with our findings in VHL-HB (Fig. 1E–G, and Table 1). The anti-proliferative effects of somatostatin analogues such as octreotide have been attributed to the presence of SSTR2 and SSTR5, the SSTR2/5 ratio specifically^32^,^38^. Additionally, broader SSTR subtype expression patterns have been found for various types of tumors. For example, SSTR3, 2 and 5 are frequently present on nonfunctioning pituitary adenomas, while only SSTR2 and 5 are present on GH-secreting pituitary adenomas^37^.

Stromal cells live-cultured from surgically resected VHL- HBs demonstrated decreased survival in response to treatment with escalating doses of the somatostatin-analogue octreotide. Analogous findings of decreased cell viability have been shown with the use of octreotide and pasireotide in various types of pituitary tumors, regardless of SSTR subtype expression pattern, *in vitro*^39^. The findings of the present study suggest this may be mediated by SSTR-induced upregulation of the direct pro-apoptotic factor BAX. Similar findings have been demonstrated in pituitary and breast cancer cells^29^,^40^ possibly mediated by the phosphatase SHP-1 working in a p53-independent mechanism^41^. Further, upregulation of SSTR2 in pancreatic tumors has been associated with a direct increase of BAX expression, relative to bcl-2, mediating cellular apoptosis^30^. Consistently, 9 months of octreotide treatment in our patient led to central photopenia on DOTATATE imaging, suggesting tumor necrosis^42^,^43^. In this study, we also demonstrate that the canonical VHL pathway^25^,^26^,^27^ is not affected by SSTR activation. This suggests that agents targeting the canonical VHL pathway that have shown partial efficacy^8^,^10^ may potentiate SSTR agonist therapy, and may be reserved for instances of tachyphylaxis with somatostatin analogues^15^,^44^,^45^.

Patients with VHL disease currently have an average life expectancy of 59.4 years for men and 48.4 years for women^11^. Despite surgical advances, VHL-HBs remain a major source of mortality in this patient population^4^. Symptomatic VHL-HBs are sometimes deemed inoperable due to anatomical location or multiple comorbidities^10^. Systemic salvage therapy with anti VEGF and anti-angiogenic chemotherapeutic options, such as bevacizumab, tyrosine kinase receptor inhibitors or and thalidomide has been reported anecdocaly^10^. Although useful in limited clinical use, these chemotherapeutic agents are not ideal for long-term therapy – a necessity for VHL patients. Somatostatin analogues have been successfully used for treatment of both primary and metastatic neuroendocrine tumors (NETs) and are well tolerated for extended use^18^,^19^,^46^. For indolent NETs, somatostatin analogues are capable of inhibiting the secretory function as well as growth characteristics^36^. Caplin *et al*. and Rinke *et al*. conducted randomized clinical trials of patients with metastatic neuroendocrine tumors and showed that somatostatin analogues significantly prolong progression free survival and stabilize tumor volumes^19^,^47^. Somatostatin analogues like octreotide have also been studied in various other cancers known to express SSTRs, such as breast, prostate, gastrointestinal, and even CNS tumors such as glioblastoma and meningioma^36^. Taken together, these results suggest that somatostatin analogues may provide a long-term treatment option for the management of indolent, unresectable VHL-HBs.

The increasing life span of patients with VHL and our greater understanding of the morbidity of their repeated craniotomies for intra-axial HBs has necessitated an exploration of alternative treatment strategies^5^. Specifically, the thousands of patients with VHL studied at the NIH in a natural history protocol for CNS tumors has allowed for an understanding of the potential catastrophe of these tumors, particularly in their infrequent occurrence in surgically unrespectable locations^4^. The somatostatin-receptor expression of HBs has been theorized in the past, in particular due to the SSTR expression of VHL-null RCC and case reports of VHLs with DOTATATE-PET signal^16^,^17^. However, the reliable demonstration of SSTR expression in HBs, in addition to the clear treatment utility, raises interesting questions about a potential neuroendocrine origin of HBs. Regardless, this finding of SSTR expression and potential anti-tumor efficacy merits expansion into a clinical trial and holds the potential for altering the way we manage this debilitating disease.

Our data from off-label use of Octreotide-LAR in a 64-year-old female patient with a severe VHL phenotype showed that 9 months of octreotide treatment led to resultant clinical stability and decreased tumor volume. Although further investigation is necessary to elucidate octreotide’s mechanism of action in HBs, these results are clinically promising and warrant clinical evaluation.

## Materials and Methods

### Clinical treatment and tissue sample collection

VHL-HB samples were obtained under protocols NIH #03-N-0164, NCT00060541 (Institutional Review Board of the National Institute of Neurological Disorders and Stroke at the National Institutes of Health) from subjects undergoing clinically indicated surgical resection of symptomatic tumors^4^. All samples and methods used for this study were done so in accordance with aforementioned approved guidelines and with NIH ethical guidelines. Subjects gave informed consent for use of their tissue for research purposes and possible publication. Written informed consent for publication of their clinical details and/or clinical images was obtained from the patient in this study and a copy of the consent form is available for review by the Editor of this journal. The treatment of a single patient with octreotide was initiated with off-label indication of FDA-approved formulations of Octreotide^48^.

### Hemangioblastoma Stromal Cell Extraction

Extraction of stromal cells from VHL-HBs was adapted from Park *et al*. 2007. Briefly, sterile resected surgical specimens were harvested from VHL-HBs and were immediately submerged in warm media (1:1 Advanced MEM (Gibco, MA, USA): RPMI (Gibco. MA, USA) with 15% Horse Serum (Gibco USA), 5% Heat-inactivated Fetal Bovine Serum (Gibco USA), L-Glutamine (Gibco USA), Penicillin Streptomycin (Gibco USA). All methods henceforth performed in sterile conditions. Specimen washed 2x with Hanks Balanced Salt Solution (HBSS; Corning) and single-cell suspensions were established by mechanical disaggregation, manual trituration and enzymatic digestion utilizing Papain (Lyophyilzed, 15 U/mg activity, LS003119, Worthington Biochem, Lakewood, NJ) and DNase I (1 U/ul, D4263, Sigma USA) following manufacturer’s protocol. The cellular layer was isolated from contaminating red blood cells and cellular debris with lymphocyte separation medium (Corning) and density-gradient centrifugation. Cells were then washed 2x with HBSS, resuspended in media with the addition of 100 Units/ml of epoetin alpha (EPO) (2000 U/ml Amgen) and plated in tissue culture nonpyrogenic polystyrene treated plates (Corning).

### VHL-HB Primary Cell Culture

Cells were incubated at 37 °C and utilized for experimental procedures within 24 hours of harvest. Media: 1:1 AMEM:RPMI with 15% Horse Serum, 5% Fetal Bovine Serum, L-Glutamine, Penicillin Streptomycin, and 100 u/ml EPO if to be used for cell viability analysis.

### Immunohistochemistry

Immunohistochemistry (IHC) for SSTRs 1, 2a, 3, 4, and 5 was performed on formalin fixed paraffin embedded (FFPE) HBs from 18 different VHL patients. Banked normal cortex was used as control. IHC staining was performed on Leica Bond Max automated stainer. Antibodies used were anti-Somatostatin Receptor 1 (SSTR1) (1:125, LS-C332380, Lifespan Bio, Seattle, WA), anti-Somatostatin Receptor 2a (SSTR2a) (1:500, PA3-109, Thermo Fischer Scientific Inc., Rockford, IL), anti-Somatostatin Receptor 3 (SSTR3) (1:100, TA323276, Origene, Rockville, MD), anti-Somatostatin Receptor 4 (SSTR4) (1:500, GTX70677, Genetex, Irvine, CA) anti-Somatostatin Receptor 5 (1:50, ab109495, Abcam, Cambridge, MA).

The slides were interpreted independently by two blinded neuro-pathologists. Receptor expression was classified on a graded 5-point scale: negative to strongly positive (−, +/−, +, ++, +++) taking both staining intensity and percentage of positive cells into consideration as described here: expression considered negative (−) if no visible stain was present throughout entire specimen; positive-negative (+/−) if specimen demonstrated a weakly positive staining intensity in 30–50% of cells; positive (+) if specimen demonstrated a weakly positive staining intensity in 80–100% of cells; double positive (++) if specimen demonstrated a moderately positive staining intensity in 80–100% of cells; triple positive (+++) if specimen demonstrated a strongly positive staining in 80–100% of cells. For instances where an agreement was not achieved, a combined blinded review was performed to achieve a consensus grading.

### XTT Cell Viability Assay

Following extraction, VHL-HB stromal cells were seeded at approximately 1 × 10^4^ cells/well in a 96-well flat bottom microtiter plate. To evaluate the effects of octreotide on cell viability, cells were exposed to octreotide acetate (0–8 μg/ml. Fresenius Kabi USA, LLC, Schaumburg, IL) for 48 hours at 37 °C. At the end of the incubation period, XTT cell proliferation assay (ATCC Manassas, VA) were performed per manufacturer’s instructions, and read on an ELx800 Absorbance reader (BioTek Instruments, USA).

### RNA extraction and Quantitative real-time PCR

Total RNA was extracted with the RNeasy Mini Kit (Qiagen, USA) following the manufacturer’s instructions and cDNA was synthesized using the SuperScript III First-Strand Synthesis SuperMix (Invitrogen Life Technologies, USA). RT-PCR was performed using CFX384 Real Time System (Bio-Rad, USA) and SYBR Green Master Mix (Applied Biosystems Foster City, CA). Gene expression was then measured with CFX384 Real Time System (Bio-Rad, USA) with primer sets for endothelin-1 (EDN1, Catalog #QT00088235, Qiagen, USA), erythropoietin (EPO, forward: 5′-GCT GCA TGT GGA TAA AGC CCG-3′; reverse: 5′-CAC ACC TGG TCA TCT GTC CC-3′) (Sigma-Aldrich, USA), glucose transporter 1 (GLUT1, Catalog #QT00068957 Qiagen, USA), vascular endothelial growth factor (VEGF, Catalog #QT01682072 Qiagen, USA), Bax forward: 5′-GTT TCA TCC AGG ATC GAG CAG-3′; reverse: 5′-CAT CTT CTT CCA GAT GGT GA-3′ and (Sigma-Aldrich, USA) and Bcl2 forward: 5′-CCT GTG GAT GAC TGA GTA CC-3′; reverse: 5′-GAG ACA GCC AGG AGA AAT CA-3′ (Sigma-Aldrich, USA) with β-Actin as internal control (Sigma-Aldrich, USA) and using Power SYBR Green PCR Master Mix (Applied Biosystems Foster City, CA).

### Apoptosis Assay

VHL-HB stromal cells were plated in 100-mm dishes at about 5 × 10^5^ seeding density. Cells were then exposed to octreotide acetate at 1 or 4 μg/ml for 48 hours prior to being trypsinized, fixed in 4% formaldehyde, and permeabilized with 100% ice-cold methanol. For apoptosis assay, after fixation and permeabilization, cells were incubated with mixed anti-cleaved Caspase-3 (cC3) at 1:6400 (9664 S Cell Signaling, Beverly, MA) and anti-cleaved PARP (cPARP) at 1:800 (5625 S Cell Signaling, Beverly, MA) for one hour then stained with species-specific secondary antibody for 30 minutes. Lastly, cells were incubated in DAPI (1 μg/ml) and then analyzed by flow cytometry (MoFlo Astrios cell sorter with Summit acquisition software, Beckman Coulter Pasadena, CA). Data analysis was completed with Kaluza software (Beckman Coulter Pasadena, CA).

### VHL-HB tumor volume

The authors utilized OsiriX (v4.1.2)^49^ for serial tumor volume measurements by manual region-of-interest (ROI) assignment for tumor area on individual adjacent image slices. These ROI areas were then multiplied by slice thickness, and total number of slices with ROI added to obtain tumor volume. The patient developed mild renal failure during the study, due to continued growth of renal cell tumors, precluding gadolinium contrast administration. Tumor volumes were measured on volumetric non-contrast fluid attenuated inversion recovery (FLAIR) images (TE/TR: 335/8000 milliseconds).

### Statistical Analysis

*In vitro* studies were subject to a minimum of three independent experiments. Data are presented as mean ± SD. Statistical analysis was performed using GraphPad Prism Software (GraphPad Software, Inc., www.graphpad.com). One way ANOVA was used for comparing results of qrtPCR. Dunnett test was used to test the differences with multiple comparisons between groups. A p value of <0.05 was considered statistically significance.

## Additional Information

**How to cite this article**: Sizdahkhani, S. *et al*. Somatostatin receptor expression on von Hippel-Lindau-associated hemangioblastomas offers novel therapeutic target. *Sci. Rep.* **7**, 40822; doi: 10.1038/srep40822 (2017).

**Publisher's note:** Springer Nature remains neutral with regard to jurisdictional claims in published maps and institutional affiliations.

## Supplementary Material

Supplementary Information

## Figures and Tables

**Table 1:** A total of 9 VHL-HBs were stained against somatostatin receptor subtypes 1, 2a, 3, 4, and 5.

Tumor	SSTR1	SSTR2a	SSTR3	SSTR4	SSTR5
1	+	+++	++	+	++
2	+++	+++	—	+	+
3	+	++	—	+++	++
4	++	++	—	+/−	—
5	+++	++	—	+	++
6	+++	++	—	+	+/−
7	—	+	—	+	+/−
8	++	+	—	+/−	+
9	++	—	+/−	+/−	++
9 Total	8 Total	8 Total	2 Total	9 Total	8 Total

Results were acquired and recorded for each tumor 1–9 based on a neuropathological scale for receptor staining positivity, as described in the materials and methods. The total number of positive staining tumors are found at the bottom of the table; each column represents a somatostatin receptor subtype.
