# Supplementary material for: Somatostatin receptor expression on von Hippel-Lindau-associated hemangioblastomas offers novel therapeutic target
Source: Sci Rep. 2017 Jan 17;7:40822. doi: 10.1038/srep40822 (PMC5240113; doi:10.1038/srep40822)
Supplement: Supplementary Information [file srep40822-s1.pdf]

Somatostatin receptor expression on von Hippel-Lindau-associated hemangioblastomas offers novel therapeutic target

Supplementary File

Saman Sizdahkhani[1¶], Michael J. Feldman[1¶], Martin G. Piazza[1], Alexander Ksendzovsky[1,4], Nancy A. Edwards[1], Abhik Ray-Chaudhury[1], Dragan Maric[2], Marsha J. Merrill[1], Karel Pacak[3], Zhengping Zhuang[1] and Prashant Chittiboina[1]*

[¶]These authors contributed equally to this study.

[1]Surgical Neurology Branch, National Institute of Neurological Disorders and Stroke, National Institutes of Health, Bethesda, Maryland
[2]Flow Cytometry Core, National Institute of Neurological Disorders and Stroke, National Institutes of Health, Bethesda, Maryland
[3]Section on Medical Neuroendocrinology, National Institute of Child Health and Human Development, National Institutes of Health, Bethesda, Maryland
[4]Department of Neurosurgery, University of Virginia Health System, Charlottesville, Virginia

[*]**Correspondence:** Prashant Chittiboina: Surgical Neurology Branch, National Institute of Neurological Disorders and Stroke, National Institutes of Health, Bethesda, Maryland 20892, USA.

Supplementary Table and Figures:

Supplementary Table 1:

Tumor	SSTR1	SSTR2a	SSTR3	SSTR4	SSTR5	Total Stained
83	+	+++	++	+	++	5
3869	+++	+++	-	+	+	5
186	+	++	-	+++	++	5
3360	++	++	-	+/-	-	5
3555	+++	++	-	+	++	5
5702	+++	++	-	+	+/-	5
1203	-	+	-	+	+/-	5
4330	++	+	-	+/-	+	5
5254	++	-	+/-	+/-	++	5
3044	+	+++		+	+++	4
2212	++	++	-	+/-		4
456	++	+/-	+++	++		4
3643	++	+/-	++	+/-		4
1837	+	+	-	+		4
3039	+++	+	+/-	+++		4
5643	++	-	+	-		4
5285	++		++	+	+++	4
5731	+/-		+	-	++	4
6603	++		-	+/-	+/-	4
1726	-		-	-		3
4920	+			+	-	3
5421	+			-	+	3
5595	+++		-	+		3
6171	-			++	++	3
4998	+			+		2
831B		+++				1
562					+++	1
27 Total	17/26 total	14/17 total	8/20 total	21/25 total	15/17 total	

A total of 27 VHL-HBs were utilized for IHC staining against SSTRs. Specimen size limited complete staining of all receptor subtypes (1, 2a, 3, 4, and 5) for all tumors. Available results of receptor staining positivity recorded above. Each column represents a somatostatin receptor subtype. Total number of positive tumors with respect to total tumors stained for each subtype are recorded at the bottom of the table.

Supplementary Figure 1:

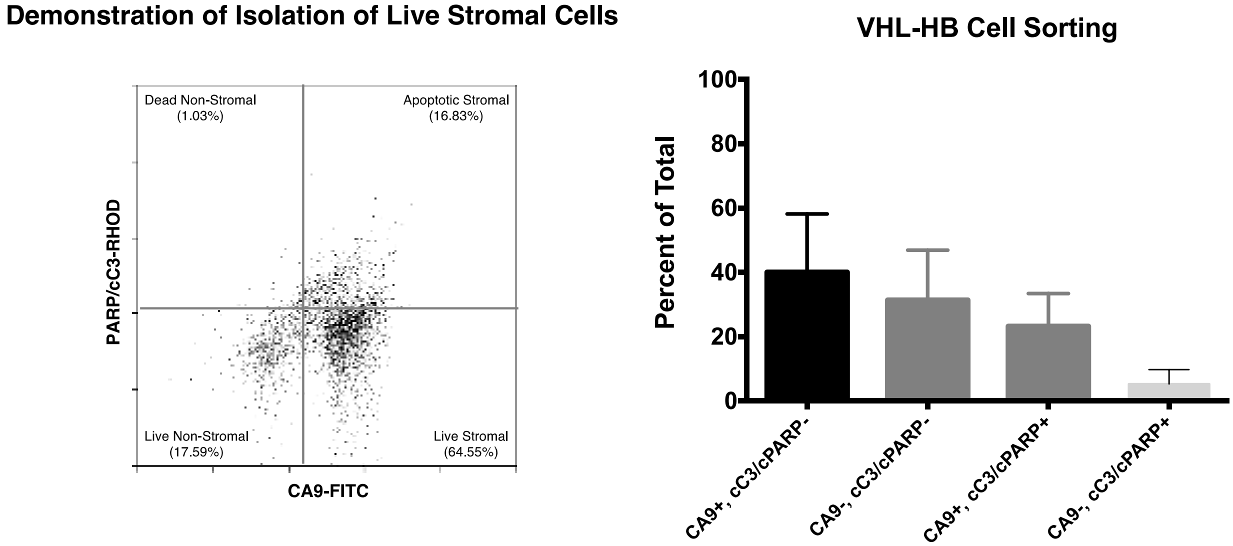

Validation of stromal cell extraction from surgically resected VHL-HBs. Representative panel of flow cytometry results. Cells were first stained for stromal cell marker CA-IX and apoptotic markers cleaved Caspase-3/cleaved PARP (cC3/cPARP). Cells positive for CA-IX and negative for cC3/cPARP indicated live stromal cells. Averages of three separate experiments, conducted in technical triplicates are shown.

Supplementary Figure 2:

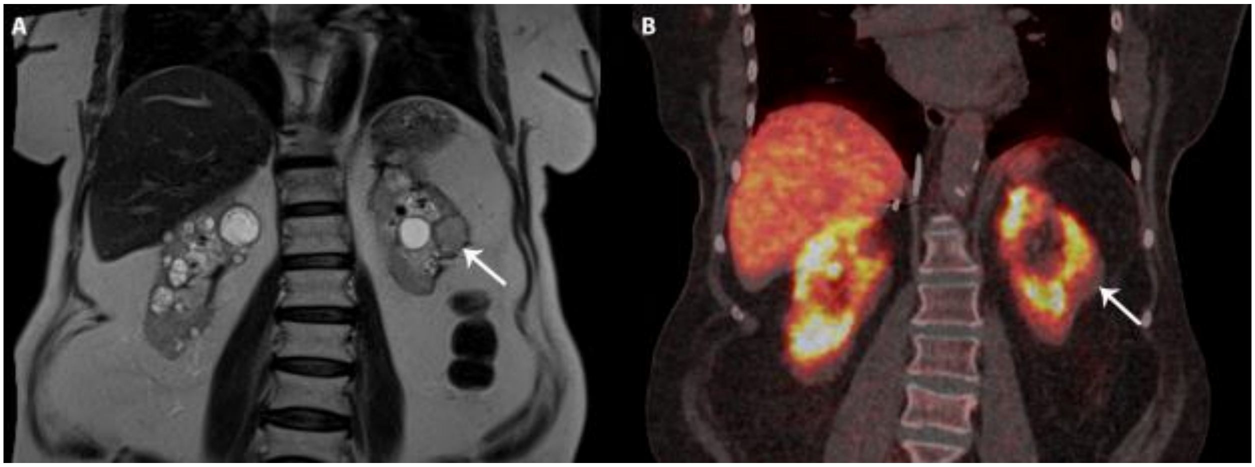

VHL-associated clear cell renal cell carcinoma (ccRCC). (A) T1 magnetic resonance image with contrast of 64 year-old female patient with germline *VHL* missense mutation presents clear cell renal cell carcinoma (arrow). (B) ^{68}GA-DOTATATE PET/CT imaging of kidneys does not show ^{68}GA-DOTATATE uptake (arrow).
